# Vertebral arteriovenous fistulae (AVF) and vertebral artery aneurysms in neurofibromatosis type 1: A case report and a systematic review

**DOI:** 10.1097/MD.0000000000030952

**Published:** 2022-10-07

**Authors:** Jiali Zhao, Guangyu Zhao, Lin Lu, Chunxia Li, Ruirui Yang

**Affiliations:** a Department of Neurology, Shandong Provincial Hospital Affiliated to Shandong First Medical University, Jinan 250021, Shandong, China; b Department of Neurosurgery, Shandong Provincial Hospital Affiliated to Shandong First Medical University, Jinan, Shandong, China.

**Keywords:** neurofibromatosis type 1, vertebral arteriovenous fistulae (V-AVF), aneurysms, endovascular intervention

## Abstract

Neurofibromatosis type 1 (NF1) is caused by mutations in the NF1 gene on the long arm of chromosome 17, which affects the skin, nervous system, eyes, and skeleton system. Vertebral arteriovenous fistula (AVF) associated with neurofibromatosis type I (NF-1) is rare.

**Case presentation:**

We report a 31-year-old postpartum woman with NF1 with vertebral arteriovenous fistulae (AVFs). She presented to our hospital because of neck pain, intracranial hypotension headache, and right upper limb weakness. She had a family history of NF1. After endovascular intervention, the AVF disappeared. However, a new aneurysm appeared on the right vertebral artery V5 dissection after 6 months of follow-up.

**Conclusions:**

The presence of NF1 in patients who present with neurologic signs should prompt further angiography. Awareness of the coexistence between NF1 and AVF or aneurysm is crucial to avoiding diagnostic delays. Endovascular occlusion of VV-AVF in NF-1 patients is effective and safe.

## 1. Introduction

Neurofibromatosis 1 is an autosomal dominant genetic disorder with an incidence of 1 in 3000 to 4000.^[1]^ The coexistence of neurofibromatosis 1 (NF1) with arteriovenous fistulae (AVF) and vertebral artery (VA) aneurysms is rare. Whether vertebral AVF in NF-1 is innate or NF-1 disease progression is still unclear, and there are no guidelines for the best treatment to date. We reported a case of vertebral arteriovenous fistula with NF1 and a new vertebral artery. Herein, a description of this case and a systematic review are presented. We summarized the clinical features and tried to explore and illustrate the underlying mechanism.

## 2. Case presentation

A 31-year-old woman presented to our hospital with neck pain for approximately one month, headache for 20 days, and right upper limb weakness for 7 days. She had a 22-day history of cesarean section. There was no history of hypertension, diabetes mellitus, or cardiac arrhythmia.

On physical examination, she was afebrile, with a blood pressure of 134/94 mm Hg. She had clear consciousness. The neurological examination indicated normal cranial nerves and neck stiffness. The right upper limb muscle strength was at level 4, and the right Babinski sign was positive. There was no sensory loss or cerebellar signs. There were numerous subcutaneous neurofibroma and café-au-lait spots on the skin, similar to her mother and grandmother.

**Figure 1.**
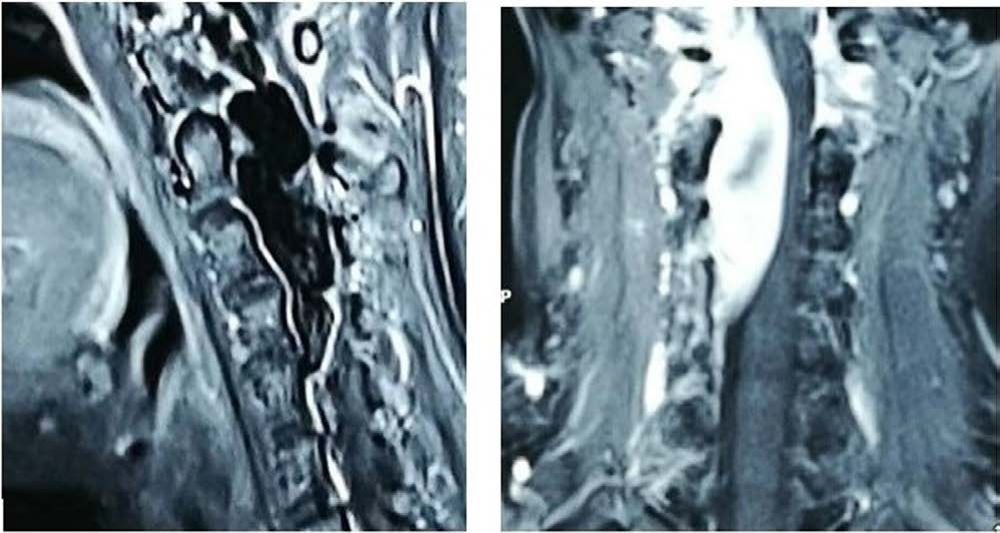
MR scan revealing an abnormal signal (5*1.2 cm) inside and outside the spinal canal at the C1-3 level. MR = magnetic resonance.

**Figure 2.**
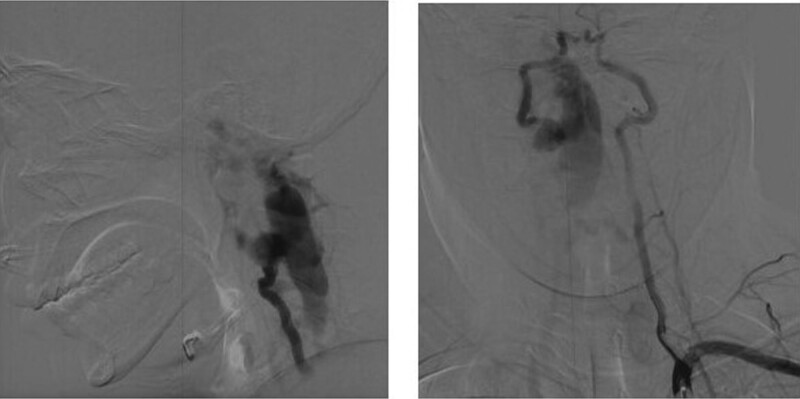
DSA prior to treatment: showing a giant arteriovenous fistula. DSA = digital subtraction angiography.

Lumbar puncture revealed that the cerebrospinal fluid pressure was lower than 50 mmH_2_O. Her gene sequencing showed that c.3916C > T in the NF1 (17q11 NM_000267.3) gene Exon29 was a heterozygous point nonsense mutation; P (Arg1306*) amino acid at the transcriptional level and premature termination of protein translation. A cervical magnetic resonance scan showed an abnormal signal (5*1.2 cm) inside and outside the spinal canal at the C1-3 level (Fig. 1). The lesion was closely related to the adjacent right vertebral artery, which was considered a vascular malformation. Then, the patient was transferred to the neurosurgical ward. Digital subtraction angiography showed a giant AVF that extended from C1 to C3 inside and outside the spinal canal (Fig. 2). Right vertebral angiogram showed an enlarged right vertebral artery V1 that drained into a massively enlarged venous plexus through a fistula at the C1 to C3 levels. Right thyroid angiography showed that the right thyrocervical and costocervical trunks were involved in the blood supply.

**Figure 3.**
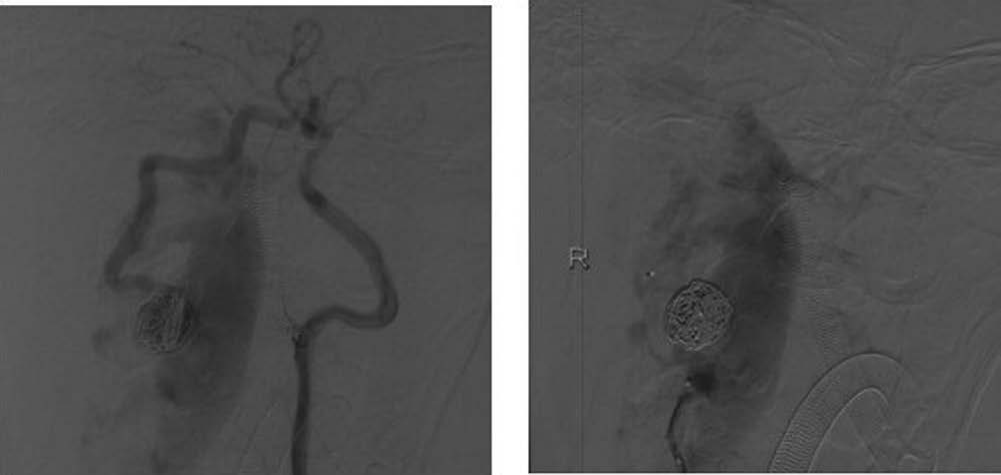
After treatment: DSA confirmed successful occlusion of the fistula. DSA = digital subtraction angiography.

The endovascular intervention strategy was to occlude the feeding arteries and varix. The 6F- and 5F-guided catheter was introduced into the right and left vertebral arteries, respectively. Two Echelon-14 microcatheters were advanced to the right vertebral artery V2 and approached to reach the fistula through a 6F guiding catheter. We deployed 7 coils altogether at the site of the fistula for the obliteration of varix through the second microcatheter (Fig. 3). Then, the AVF disappeared when Onyx glue was used under fluoroscopic control. After intervention, the subject’s symptoms improved, and she was discharged.

After the 6-month follow-up, the patient reported intermittent headache for approximately 2 months. A follow-up angiogram showed a right vertebral artery V5 issection aneurysm and no contrast filling of the initial AVF. The doctor recommended follow-up observation.

## 3. Discussion and conclusions

Neurofibromatosis 1, also called Von Recklinghausen’s disease, is an autosomal dominant genetic disorder with an incidence of 1 in 3000 to 4000.^[1]^ It is caused by mutations in the NF1 gene on the long arm of chromosome 17.^[2]^ Neurofibromin, the protein product of the NF1 gene, functions in part as a negative regulator of the p21 Ras proto-oncogene.^[3]^ Vascular abnormalities associated with NF1 are rare and may reflect disruption of the vascular maintenance and repair regulated by neurofibromin.^[4]^ There has been an increasing awareness of the association of vascular lesions in NF1, including stenoses, aneurysms, and AVFs.^[5]^

Our literature was reviewed using the PubMed database. We revealed 47 cases of vertebral arteriovenous fistula associated with NF1. Their mean age was 40.04 years (range 11–66 yr). There were 35 females and 11 males (one case unknown). Twenty-five lesions occurred on the left, 20 on the right, and one occurred bilaterally. The most common location of the fistula was in the V2 segment of the vertebral artery at the level of the C1–C6 cervical vertebrae (68.09%; 32/47). The V3 segment at the level of C1 & above was implicated in 6 lesions, and lesions below the C6 level (in the V1 segment of the vertebral artery) were less common (4.26% 2/47). AVF with concomitant aneurysms occurred in 9 lesions (19.15%, 9/47). Three female patients with AVF in NF1 were postpartum. Trauma was seen in 4 cases (4/47). Two patients reported a nonsense mutation in the NF1 (17q11 NM_000267) gene (Table 1).

**Table 1:** Summary of patient demographics and fistula characteristics.

Variables	Male	Female	NR	Total
N (%)	11	35	1	47
Age	38.91	39.88	59	40.06
Side				
Left	7	17	1	25
Right	4	16	0	20
Bilateral	0	1	0	1
NR	0	1	0	1
Level of fistula				
V1 (below C6)	1	1	0	2
V2 (C1–C6)	7	24	1	32
V3 (C1 & above)	0	6	0	6
Other	3	4	0	7
Associated aneurysm	1	7	1	9
Trauma	3	1	0	4
Postpartum	0	3	0	3
Gene (report)	1	1	0	2

Presenting symptoms of the AVF were described in 47 cases. The most common symptom was neck pain, which was seen in 25/47 (53.19%) patients. Other common symptoms were weakness in the extremities, numbness, bruit in the neck, and tinnitus (Table 2).

**Table 2:** Summary of clinical presentation of VAVF in NF1 patients.

Symptom	n
Pain	27
Motor weakness	13
Bruit	14
Swelling in neck	2
Numbness	11
Tinnitus	11
Headache	2
Bladder involvement	3
Hoarseness of voice	1
Difficulty in breathing	1
Comatose	1
Radiculopathy	4
Radiculomyelopathy	4
Scoliosis	1

Out of the 47 cases, 4 cases did not report any of the surgical/endovascular interventions, and the treatment outcome of one patient with coils was not mentioned in the article. They were also excluded from the “treatment outcome comparative analysis”. Out of the remaining 42 cases, 9 (9/42) underwent surgical treatment, and 33 (33/42) underwent endovascular treatment. After direct surgery or endovascular treatment, 88.1% (37/42) of patients had good outcomes (Table 3).

**Table 3:** Treatment intervention: outcome.

Treatment	Outcome	
	Good	Bad
Surgical (9/42)	7	2
Endovescular (33/42)	30	3
	37	5

Deans et al suggested two possible mechanisms by which AVF may arise in patients with NF-1: dysplastic smooth muscle or neurofibromatosis proliferation in the arterial wall could lead to aneurysm formation and leakage and ultimately rupture into adjacent veins; AVF could arise congenitally as a manifestation of mesodermal dysplasia.^[40]^ However, whether vertebral AVF in NF-1 is innate or NF-1 disease progression remains unclear. Yingjin Wang et al speculated that this rarely reported deformity might be a congenital disease with mesodermal dysplasia because of a novel stop codon mutation in the NF1 (NM_000267) gene exon 4.^[6]^ However, we think it is controversial to speculate that AVF is a manifestation of mesodermal dysplasia by mutation in the NF1 gene.

In our case, the AVF extended from C1 to C3 inside and outside the spinal canal. Gene sequencing showed that c.3916C > T in the NF1 (17q11 NM_000267.3) gene Exon29 is a heterozygous point nonsense mutation. The mutation led to the termination of the 1306th amino acid at the transcriptional level and premature termination of protein translation. This variant has been reported to be detected in a sporadic NF1 patient, but that research did not report AVF. Our patient, her mother and grandmother had neurofibromas on the skin, but her mother and grandmother had no neurological symptoms. Furthermore, it is interesting that our patient presented in the postpartum period, possibly with hemodynamic factors related to hormonal changes and blood volume. After intervention with coils and Onyx glue, the AVF of our patient disappeared, but a vertebral artery aneurysm appeared again. We believe NF-1 may sometimes present as abnormalities of connective tissue that result in vessel vulnerability and friability, and the vertebral artery can be subjected to significant shear stress in the cervical spine as it is tethered to the foramen transversarium of C6 to C1. The combination of shear stress with vessel dysplasia may predispose NF-1 patients to develop a high-flow VV-AVF spontaneously or following minimal injury.^[41]^ The lack of neurofibromin, which is expressed by the NF-1 gene, might promote the pathological process.^[42]^ In summary, we think AVF associated with NF-1 is more likely to be a secondary factor resulting in vessel vulnerability and friability.

Clinicians should consider vertebral arteriovenous fistula in NF1 patients with neck pain and numbness. The presence of NF1 in subjects who present with neurologic signs should prompt further angiography. Awareness of the coexistence between NF1 and AVF or aneurysm is crucial to avoiding diagnostic delays. In conclusion, endovascular occlusion of VV-AVF in NF-1 patients is effective and safe.

## Author contributions

**Conceptualization:** Jiali Zhao.

**Data curation:** Jiali Zhao, Guangyu Zhao.

**Formal analysis:** Jiali Zhao, Guangyu Zhao, Lin Lu, Chunxia Li, Ruirui Yang.

**Investigation:** Jiali Zhao.

**Methodology:** Jiali Zhao, Guangyu Zhao.

**Project administration:** Jiali Zhao.

**Writing – original draft:** Jiali Zhao.

**Writing – review & editing:** Jiali Zhao, Guangyu Zhao, Lin Lu, Chunxia Li, Ruirui Yang.
